# Supplementary material for: Are carbon emissions trading and green financial instruments synergistic? -Comprehensive quantitative research based on content analysis
Source: PLoS One. 2024 Mar 7;19(3):e0298601. doi: 10.1371/journal.pone.0298601 (PMC10919601; doi:10.1371/journal.pone.0298601)
Supplement: S1 Data — (ZIP) [file pone.0298601.s001.zip › Data/地级市Z_F工作报告.zip!/░▓╗╒/║╧╖╩╩╨/└┤╫╘╣1⁄2╓┌║┼í╛┬φ┐╦╩2╛▌═°í┐_2010.docx]

# **2010年政府工作报告**

发布时间：2010-01-19 15:01

  各位代表：
                           
                           　　现在，我代表市人民政府，向大会作政府工作报告，请予审议，并请市政协委员和其他列席人员提出意见。
                           
                           　　一、2009年工作回顾
                           
                           　　2009年是合肥发展极不平凡的一年。既是建国60周年大庆之年，也是我们经受国际金融危机重大考验，在逆境中奋力崛起的一年。一年来，在省委、省政府和市委的坚强领导下，全市人民以深入学习实践科学发展观为动力，凝心聚力，化危为机，继续强力推进"大发展、大建设、大环境"，全面贯彻落实中央及省保增长保民生保稳定各项决策部署，再次取得可喜的发展业绩，实现了又好又快的发展，经济总量再上新台阶，城市辐射带动力进一步增强，人民生活不断改善，社会保持和谐稳定，市十四届人大二次会议确定的年度目标任务全面超额完成。
                           
                           　　预计，全市地区生产总值突破2000亿元，增长17%以上，省内经济首位度提升至20%以上。财政收入341.9亿元，增长13.5%，其中地方收入180.9亿元，增长12.4%。全社会固定资产投资2468.4亿元，增长34.3%。社会消费品零售总额703.7亿元，增长19.6%。城镇居民人均可支配收入17158元，农民人均纯收入6065元，分别增长10.1%和13%。城镇登记失业率4.05%。单位GDP能耗下降率及化学需氧量、二氧化硫排放削减量均在省控目标以下。
                           
                           　　一年来，主要工作有：
                           
                           　　（一）积极应对金融危机，保增长举措有力、成效显著
                           
                           　　面对国际金融危机的冲击，我们坚定信心，主动作为，紧密结合合肥实际，及时出台了一系列保增长政策措施，有效地化解金融危机的影响，实现了经济回升向好。
                           
                           　　集中财力保增长。大力压缩行政性经费，实现车辆购置零增加，出国、会议费用分别降低40.74%和8.6%；积极推进市直行政事业单位日常办公用品、宣传、印刷等定点采购，资金节约13.4%。加大财政资金奖补力度，实行窗口受理、预拨兑现，重点支持工业、外贸企业扩大产销，支持自主创新和新兴产业培育。全年用于支持工业发展资金26.6亿元，各类企业均受到支持；免收开发园区、乡镇工业园2305家企业各种行政性费用3.59亿元；支持科技创新及产业结构优化升级资金8.86亿元。
                           
                           　　狠抓项目建设保增长。紧抓扩内需政策机遇，累计争取国家及省项目资金16.2亿元。全年新开工项目3532个，同比增加1390个，其中新开工亿元以上项目193个，省"861"、市"1346"行动计划项目分别完成投资765亿元、721亿元。全年完成工业投资752.2亿元，增长40.4%；熔安动力首台机下线，海尔冰箱二期、格力电器一期等项目建成投产，京东方六代线、大陆轮胎一期、江汽两万辆客车基地、三洋机电产业园等开工建设。房地产市场平稳健康发展，全年完成商业及住宅投资539.02亿元，增长10.4%；商品房销售面积 1298万平米，增长40.8%。
                           
                           　　全力服务企业保增长。进一步健全联系重点企业及大企业现场办公会等制度，再次选派120名市直机关干部驻企帮扶。加强煤电油运等生产要素的统筹调度，开展工业园区市政基础设施建设的对接服务。搭建大宗工业品、消费品供需对接平台，认真落实家电、汽车摩托车下乡及以旧换新等政策，支持骨干企业开拓市场，全年累计补贴家电及汽车摩托车下乡资金5136万元。
                           
                           　　强化银政企合作保增长。积极推介优质企业，完善考核奖励办法，引导金融机构扩大信贷投放，促进银企互利双赢；支持商业银行建立小企业金融服务专营机构；举办季度银企对接会，累计签约项目395个、贷款额175.4亿元；全年新增贷款924亿元，增长35%。鼓励发展股权质押、动产质押、仓单质押等新型融资方式，发行两期、总规模3亿元的"滨湖·春晓"中小企业集合信托计划；三县农村信用社改革全部完成，小额贷款公司试点达19家，新增典当行40家；发行企业债券40亿元，安科生物、皖通科技成功上市，新华传媒通过上市审核。
                           
                           　　（二）坚持创新驱动，工业立市不断取得新进展
                           
                           　　深入推进合芜蚌自主创新综合配套改革试验区和科技创新型试点市建设，加快战略性新兴产业培育和主导产业技术改造，大力推进经济发展方式转变和产业结构调整。
                           
                           　　强化创新平台建设，优化创新机制，创新能力显著提升。全市规模以上工业企业全部设立研发机构，新增国家级企业技术中心3家、省级12家。新增国家级自主创新产品6个、省级创新型企业23家，专利授权量超过2100件，新认定高新技术企业109家。中国风险投资研究院合肥分院、安徽股权交易所挂牌，各类风险投资公司发展到20家。成立合肥"科技路路通"分中心，建立汽车自动化装备、环保动力能源等产业技术创新战略联盟。示范区及核心区建设提速，"一个中心、三个基地" 创新平台建设加快推进，大学科技园、科研孵化基地等项目全面开工。
                           
                           　　新兴产业培育加速，主导产业提档升级，工业总量大幅提升，结构不断优化。编制实施电子信息、新材料、软件、节能环保、公共安全等八个新兴产业发展规划。鑫昊PDP显示器、彩虹玻璃基板等项目开工建设，新型平板显示产业基地初见端倪；组建公共安全技术研究院，建设公共安全产业基地。全年高新技术产业产值1400亿元，增长30%；实现增加值约420亿元，占全市GDP比重21%。主导产业技术改造步伐加快。全年工业技改投资351.2亿元，占工业总投资46.7%，安利、应流集团等企业技改项目加快实施，安凯新能源汽车示范推广及马钢（合肥）高炉喷煤改造等工程进展顺利。全市规模以上工业企业达到1761户，增长29%；规模以上工业实现产值2749.2亿元，增加值767.5亿元，增长27.2%；汽车、家电等八大产业增加值增长27.1%，对全市工业增长的贡献率达到68.1%。
                           
                           　　坚持"双轮驱动"，促进服务业与工业融合发展，现代服务业亮点纷呈。引进中远物流、普洛斯物流等一批国内外知名物流企业，支持徽骆驼、安泰物流等企业做大做强。启动动漫及软件服务外包产业基地建设，动漫产业快速发展，企业发展到60多家、产值1.5亿元以上；服务外包企业72家，接包合同签约1.9亿美元。引进万达广场等重点项目，加快发展连锁经营、高端百货等新型流通业态，继续推进"农超对接"和"双百市场"工程。汇丰银行、进出口银行、华夏银行、九江银行、中安财险等纷纷落户合肥，合肥国际金融后台基地开工建设。旅游业发展有新突破，创建紫蓬山等3个国家4A旅游景区，中青旅、合肥旅行社晋升"全国百强社"，合肥科教游、乡村游成知名品牌，动漫主题公园成功签约。举办自主创新要素对接会、家电博览会、苗交会等各类大型会展132场，会展经济效应进一步扩大。
                           
                           　　（三）统筹城乡发展，县域经济和新农村建设呈现新气象
                           
                           　　坚持以城带乡、城乡融合统筹发展。长丰县与新站区共建平板显示产业基地，肥东龙岗开发区整体移交瑶海区，肥西县分别与高新区、经开区联合建设工业园区，县区合作开发取得新突破；完成合水路、合店路一期改造等工程，城乡公用及基础设施对接继续深入推进。全年财政拨付涉农资金5.4亿元，累计发放各类财政补贴农民资金12.7亿元。三县实现GDP470亿元，增速高于全市约1个百分点，县域经济对全市增长的贡献率达24%。
                           
                           　　城乡一体化综合配套改革试点工作扎实推进。扩权强县、土地经营权流转、综合产权交易、"双置换"等改革稳步实施，累计流转土地72万亩，占承包耕地23%。万亩土地复垦、宅基地整理和整村推进新农村建设工程成为全国样板，新增耕地4万亩。"十镇百村"工程市级示范点扩大到152个，缩并自然村1028个，近8万农民转为市民。新增农民专业合作组织230家。"农民向社区集中、土地向规模经营集中、产业向园区集中"的发展格局加快形成。
                           
                           　　农村产业结构调整步伐加快。县域工业总量进一步扩大。汽车零部件、现代建材、食品加工等产业加速发展，涌现出鸿路钢构、中南光电、亿恒汽配等一批支柱型企业，县域规模以上企业756家，实现增加值198.7亿元，增长46.5%；县区工业园区及乡镇工业聚集区发展到41个，桃花工业园成为全省首个税收突破10亿元园区，工业对县域经济的贡献率达65%。
                           
                           　　现代农业继续加快发展。全市高效特色农业基地超过100万亩，新增农业园区100个、设施农业5万亩、露地蔬菜10万亩、市级以上特色村镇100个，肉、蛋、奶、水产品产量68万吨；新建各类规模养殖场280多个，规模养殖比重达到75%。新认定无公害农产品基地5.8万亩，新增中国名牌产品2个、省著名商标8个。和诚肉鸡深加工、现代牧业、香港宝石集团及粮食物流园区等骨干项目加快建设，全市农业产业化龙头企业实现销售收入突破300亿元。
                           
                           　　农村基础设施建设进一步强化。实施40座中小型水库除险加固工程，解决17万人饮水安全问题；完成农村公路建设投资6.7亿元，全市行政村水泥（沥青）路通达率100%，行政村班车开通率98.3%。
                           
                           　　（四）推进重大基础设施建设，生态宜居城市建设迈出新步伐
                           
                           　　区域综合交通枢纽建设加速推进。铁路大建设如火如荼，合肥火车站改造、宁西复线、合蚌、合福客运专线、南站迁建、铁路枢纽南环线及南客站开工建设，商杭客运专线、华东二通道电气化等项目前期工作顺利推进。新桥国际机场建设工程全面推进。派河熔安动力专用码头建成，合肥港综合码头一期工程进展顺利，巢湖及裕溪复线船闸开工建设。合六路延伸段、疏港公路建成通车，城市出入口进一步通畅便捷。长江西路、裕溪路、南北一号线等三座高架桥及轨道交通一号线试验段全面开工。
                           
                           　　滨湖新区建设全面推进，全年完成投资121.9亿元，房建开工面积429万平方米，竣工面积226万平方米；滨湖轮滑场竣工，国际创新展示馆、渡江战役纪念馆、国际会展中心、安徽名人馆等馆群开工建设，成为国家"城市生态建设示范区"。政务文化新区商业、教育等配套功能不断完善，合肥大剧院投入使用，市青少年活动中心主体结构封顶。旧城改造与新区开发同步实施。畅通一环、改造二环等路桥工程加快推进。启动坝上街、粮食二库、十五里河中段片区等改造。实施景观整治工程，重点整治道路42条，建筑2915栋，老旧小区20万平米，建筑立面280万平米。
                           
                           　　城市生态环境加速提升。坚持大力度投入、高标准建设，加快推进水环境治理。继续实施巢湖沿岸生态环境综合治理，完成湖崩试验段主体工程；完成四里河、板桥河综合治理及二十埠河、塘西河、十五里河主要截污工程，铺设污水管网377公里；建成蔡田铺一期、十五里河、小仓房污水处理厂，日新增污水处理能力20.2万吨。清溪路垃圾填埋场综合治理工程获"中国人居环境范例奖"。启动城市景观水体治理试点，建立河道生态补水机制，巢湖西半湖及南淝河、十五里河、派河水质日趋改善。加大扬尘污染控制和机动车尾气污染治理力度，全年空气质量优良天数317天，比上年增加56天。危险固体废物处置中心二期工程基本建成。开展四里河、板桥河沿岸、高压走廊等廊道绿化建设，新增城市园林绿地1.1万亩；"清洁家园、绿化乡村"活动取得实效，基本完成外环森林生态长廊二期工程，全市农村植树造林3.3万亩。
                           
                           　　市政公用设施逐步完善。新建供水管网400多公里，董铺水源厂扩容改造完工，六水厂一期工程并网运行。新增天然气用户12万户，日最高供气量突破80万立方米。长江中路、长江东大街BRT公交专用道及中央岛式站台投入使用。供电、供气、供热等服务保障能力有新的提高。
                           
                           　　（五）深入推进重点领域改革开放，加快发展再添新活力
                           
                           　　围绕探索建立大部门体制、建设服务型政府，积极稳妥实施市级政府机构改革。推进市直机关公务员转任立法，建立交流轮岗长效机制。美菱、荣事达、氯碱、合钢等企业国有产权退出重组加速推进，市属化工企业整合重组深入实施，目标考核、收益收缴、企务公开等国资监管制度有效落实。非公经济较快发展，全年新增私营企业1.19万户，注册资本963.7亿元。推行绩效预算，强化行政事业单位资产管理，县（区）会计集中核算全面向国库集中支付转轨。文化体制改革取得新突破，合肥报业集团挂牌，《合肥日报》正式创刊。招投标管理体制改革向纵深推进，全年各类交易项目3334个，交易额突破400亿元，增长69.9%。工商系统市场管办脱钩工作基本完成。城市管理体制改革稳步推进，建筑垃圾运输车辆密闭改造和生活垃圾一级收运方式改革取得新进展。节约集约用地试点工作有效推进，新一轮土地利用总体规划大纲获批，闲置土地清理和违法用地专项查处取得阶段性成果。
                           
                           　　招商引资取得新突破。作为核心城市，积极参与编制皖江城市带承接产业转移示范区建设规划。组建第六批招商小组391个，发挥"合肥之友"等招商平台作用，开展"阳光地产"、"阳光市场"及国有产权转让等系列推介活动。大力度开展与央企对接合作，达成在肥投资意向项目82个，总投资额2000多亿元。全年实现招商引资1040亿元，增长33%；实际到位外资13亿美元，增长8.3%，新落户世界500强外资企业5户。
                           
                           　　对外合作进一步拓展。编制合肥经济圈和合淮同城化总体规划并付诸实施，与南京、南昌、佛山及巢湖、阜阳等地签署区域合作协议。加密国内航线、实现台北直航，开通首尔定期航班，扩大与奥斯纳布吕克市等国际友城的合作交流。
                           
                           　　承办第四届中博会取得圆满成功。到会中外客商3.8万人，参展境内外企业1000多家；签约外资项目127个、投资总额76.1亿美元，内资项目356个、投资总额1682.4亿元。被誉为是一次"盛况空前、成果丰硕、精彩纷呈、引人瞩目"的经贸盛会，集中展示了合肥乃至安徽改革开放、加速崛起的新风采。
                           
                           　　（六）坚持不懈惠民生，社会事业迈上新台阶
                           
                           　　注重民生改善和社会和谐，集中实施36项民生工程，各级财政投入资金32亿元，惠及城乡居民450余万人。
                           
                           　　社会保障体系进一步完善。农村低保提标扩面，将1988年10月前被征地农民全部纳入保障范围；新农合参合率达97.54%，住院费用补偿提升至47%；新建、改建乡村卫生服务机构82处；"515敬老工程"覆盖全部乡镇，荣获"中华慈善突出贡献奖"。建成社区示范卫生服务中心和服务站73所；35万在肥高校大学生全面纳入城镇居民医疗保险；向城区2.8万名80岁以上老人发放高龄津贴；新建、续建廉租住房29万平米，发放补贴1830万元，低收入住房困难家庭实现应保尽保。为民服务全程代理网络全面建立，惠民直达工程试点成效明显。
                           
                           　　就业再就业工作扎实推进。积极开展创建国家级创业型城市工作，推行农民工培训券制度，新建农民工创业园10个；实施"零就业家庭"援助项目，提供就业岗位1485个；全年下岗失业人员再就业2.97万人，新增城镇就业10.96万人。
                           
                           　　教育卫生文化体育事业发展加快。实施中小学校舍安全工程，开展义务教育学校绩效工资改革，义务教育均衡发展进入全国先进城市；出台扶持政策，引导学前教育健康发展；推进资源整合，高中教育水平明显提高；强化职教基地建设，合肥职教城签约入驻院校17所；合肥学院成为省级示范应用型本科高校，合肥老年大学获全国先进称号。扩充优质卫生资源，市滨湖医院、省心脑血管病医院建成开诊，市二院新区、妇幼保健院东区开工建设；加强重大疫情和传染病防控，甲型H1N1流感和手足口病防治工作平稳有效；开展肥西县、庐阳区基层医药卫生体制综合改革试点。流动人口管理、出生人口性别比治理等取得新突破，低生育水平保持稳定。建成10个乡镇综合文化站、207个农家书屋；数字电视整体转换43万户，广播电视综合覆盖率100%。第四届全国体育大会筹备工作进展有序，"全民健身、健康合肥"活动蓬勃开展。
                           
                           　　精神文明创建活动扎实推进。开展"不文明行为’评·议·改’"等系列主题活动，实施交通秩序集中整治；应急和社区网格化管理不断完善，城市管理水平进一步提升。第四次荣获"全国创建文明城市工作先进市"称号。
                           
                           　　切实维护社会和谐稳定。严厉打击各种犯罪活动，出台重大事项社会稳定风险评估办法，综治工作向民企、民医、民校拓展，治安防控体系进一步完善，连续四届荣获中国社会治安综合治理优秀城市称号，再次获得"长安杯"。严格落实信访工作各项制度，扎实开展"信访积案化解年"活动。全面排查整治安全隐患，全市亿元GDP生产安全事故死亡率较去年下降13.6%。民族宗教工作成效显著，荣获全国民族团结进步模范市称号。大力开展国防教育，扎实推进后备力量建设，重视做好双拥共建工作。积极发展妇女儿童、老龄和残疾人事业，圆满完成城市社区两委换届直选，统计、人防、档案、气象、地方志、防震减灾等进一步加强，工会、共青团、文联、科协、社联、侨联、工商联、爱卫会、红十字会等工作取得新成绩。
                           
                           　　过去的一年，我们还坚持强化政府自身建设。深入开展学习实践科学发展观活动，紧紧围绕"解放思想求创新，转变职能促崛起"主题，大力弘扬求真务实的工作作风，着力破解制约科学发展的突出问题，努力化解群众反映的难点、热点问题，基本实现了"干部受教育、发展上水平、群众得实惠"的总要求。坚持依法行政，建立政府常务会议学法制度，继续开展规范性文件备案审查及清理，试点推行行政处罚自由裁量权规范行使工作。建立人大代表列席市政府常务会议制度，自觉接受人大依法监督、政协民主监督，全年办理市人大代表议案、建议174件、市政协委员提案417件，办复率均为100%。深入开展小金库治理、村（居）"三资"清理，巩固反腐倡廉制度建设成果。认真落实"双百"制度，推进领导干部任期经济责任审计、大建设项目跟踪审计，圆满完成本级预算执行审计，并对社会公告审计结果。
                           
                           　　各位代表，2009年，是新世纪以来形势最为复杂、发展最为艰难的一年，合肥经济社会发展取得这样的成绩极其不易。这是党中央、国务院，省委、省政府和市委正确领导的结果，是市人大依法监督、市政协民主监督、社会各界大力支持的结果，是全市人民坚持加快崛起信念、锐意改革、顽强拼搏的结果。在此，我代表市人民政府，向全市广大工人、农民、知识分子和各级干部，向驻肥人民解放军指战员、武警官兵、公安干警和中央驻肥单位，向关心和支持政府工作的各民主党派、工商联、各人民团体和社会各界人士，表示衷心的感谢！
                           
                           　　回顾过去，成绩固然令人欣慰，但总体来看，发展不足、综合竞争力不强仍是我们面临的主要矛盾。特别是当前，还存在一些亟待解决的突出矛盾和问题，主要是：企业数量偏少、体量不大，后续牵动性大项目不多，战略性新兴产业尚在培育，加速发展的基础有待进一步巩固；土地等资源要素供给矛盾日渐突出；制约科学发展的深层次体制性障碍尚需大力破除；政府职能需要进一步转变，作风不实、服务意识淡漠，形式主义、官僚主义等现象还不同程度的存在。对此，我们将采取更加切实有效的措施努力加以解决。
                           
                           　　二、2010年工作目标和主要任务
                           
                           　　今年是"十一五"发展的收官之年，做好2010年的经济社会发展工作，对保持合肥加速发展之势，具有十分重要的意义。虽然宏观环境不确定、不稳定因素依然很多，但总体上看，今年的发展形势要明显好于去年，世界经济出现企稳回升的积极迹象；我国经济整体向好态势不断巩固，国家继续实施积极的财政政策和适度宽松的货币政策，在保持经济平稳较快发展的同时，加快经济结构调整、促进经济发展方式转变，在发展中促转变，在转变中谋发展；特别是国家正在加快实施中部崛起战略规划，鼓励自主创新，扩大民生投入，这些都有利于我们进一步发挥优势、抢抓加快发展的机遇。尤其是省委、省政府对合肥的发展寄予厚望，强力推进合肥经济圈、合芜蚌自主创新综合试验区和皖江城市带承接产业转移示范区建设，为我们把握大局、加快发展进一步明确了方向。
                           
                           　　就自身而言，经过近五年的大发展，合肥的工业化率达到40%，人均GDP突破5000美元，一大批重大项目相继建成，支撑经济社会持续快速发展的内生动力十分强劲，已处于新一轮经济增长周期的上升期。同时，全市上下业已形成的勇于创新、合力攻坚、加快崛起的昂扬奋进精神，正在成为我们实现"弯道超越"的强大动力。我们有理由对合肥的未来发展充满信心！
                           
                           　　2010年政府工作的总体要求：全面贯彻党的十七大、十七届三中、四中全会和中央经济工作会议精神，认真落实省委、省政府和市委的战略部署，深入实践科学发展观，继续坚持被实践证明是行之有效的既定战略和政策，继续保持开拓进取的精神状态，进一步解放思想、深化改革，以重点项目建设为主要抓手，以战略性新兴产业培育和经济发展方式转变为突破口，突出自主创新和结构调整，强化城乡统筹，着力推进社会建设，努力推动经济社会又好又快发展，确保全面完成"十一五"发展奋斗目标。
                           
                           　　2010年全市经济社会发展的主要预期目标：地区生产总值增长12%以上；财政收入增长12%；实际利用外资增长10%；社会消费品零售总额增长17%；城镇居民人均可支配收入增长8%；农民人均纯收入增长9%；城镇登记失业率控制在4.3%以内；人口出生率控制在11‰以内；居民消费价格指数103左右；单位GDP能耗下降率及主要污染物减排量达到省控目标。
                           
                           　　实现上述目标，今年要着重做好以下八个方面工作：
                           
                           　　（一）继续扩大有效投入，不断增强加快发展的后劲
                           
                           　　以国家产业政策为导向，以先进制造业、战略性新兴产业、现代服务业等为主攻方向，继续做大投资总量，优化投资结构，加快重大项目建设，为经济发展提供强有力的支撑。
                           
                           　　加快研究、谋划、储备一批牵动性强的大项目，特别是超50亿元，甚至超100亿元的大项目，努力形成持续的投资后劲。进一步健全重大项目调度、重点项目领导分工联系等制度，建立项目审批、用地指标、环境评价绿色通道，加快在建项目进度。集中力量推进京东方六代线、熔安动力、格力电器二期、三洋机电产业园、大陆轮胎一期、中盐化工等重大项目建设；积极推进京东方八代线、巢湖水环境综合治理、长安30万辆微型汽车、江汽重卡及发动机、中烟醋酸纤维、合肥电厂#6机组等重大项目前期工作。实行项目全程跟踪、全面落实、全速推进，确保早落地、早开工、早投产。
                           
                           　　坚持多资并举，努力拓宽投资渠道。继续积极利用有利的宏观环境，加大争取中央和省投资力度，发挥好政府投资引导作用。进一步加强与各类金融机构的对接合作，加大金融开放创新力度，综合运用考核激励、风险补偿等多种方式，引导金融机构持续扩大信贷投入。加强后备资源培育，适度扩大企业债券发行规模，支持企业上市和再融资。大力引进境内外创投资本，重点支持处于初创期、种子期的中小企业和科技企业发展。深入推进与央企合作对接，加快推进皖江城市带承接产业转移示范区建设，加强与长三角地区对接，有序承接国内外产业转移。进一步降低准入门槛，积极鼓励引导民间投资，激发民间发展活力。
                           
                           　　（二）以自主创新为引领，推进结构调整和发展方式转变
                           
                           　　强力推进合芜蚌自主创新综合试验区和科技创新型试点市建设，推动工业结构调整和提质升级，加快科技优势向竞争优势转变，加快推进国家创新型城市试点建设。
                           
                           　　强力推进自主创新示范核心区及"一个中心、三个基地"建设。加快资本、人才、技术等创新要素集聚，加快建设科技创新公共服务和应用技术研发中心，全力打造科研、孵化基地和产业基地。创新产学研合作模式，围绕产业技术创新链，加快建设一批产业技术创新战略联盟，突破产业发展的技术瓶颈。大力培育和发展科技中介机构、技术经纪人，开展各类科技创新平台建设。
                           
                           　　突出战略性新兴产业的培育和发展。重点支持以京东方为代表的新型平板显示产业，以四创电子、美亚光电、科大立安、量子通信试验网等为代表的公共安全产业，以江汽集团、国轩高科等为龙头的新能源汽车产业，以三洋、格力电器、阳光电源等为代表的节能环保产业，以安科生物、赛真拜通等为代表的生物产业，以及以乐凯、杰事杰等为代表的新材料产业建设。积极组织遴选一批新的重大技术创新和产业化项目，力争尽快突破若干共性关键技术，形成一批极具增长潜力的新兴产业集群。全年高新技术企业和创新型企业发展到710家，产值达到1800亿元，增加值达到550亿元，增长30%以上。
                           
                           　　大力推进产业结构优化升级。继续壮大支柱产业。围绕国家产业振兴规划，加快推进汽车、家用电器、装备制造等传统主导产业技术进步，促进产业链向高端发展、向下游延伸，形成以骨干企业为主体，与专、精、特、新中小科技型企业相配套的产业集群。以"双千工程"为抓手，依托信息技术，加快重点行业、重点企业技术改造升级。
                           
                           　　积极推进节水型社会建设，大力发展循环经济和以低能源、低污染、低排放为基础的低碳经济，在全社会倡导低碳生活方式。推进节能减排技术应用，组织实施20个工业、建筑、商贸等领域重点节能项目。
                           
                           　　（三）扎实推进城乡一体化建设，不断激发县域发展活力
                           
                           　　加快制定城乡一体化综合配套改革试点总体方案。发挥县域空间及劳动力等资源优势，强化城乡产业、规划、要素、管理服务统筹，加快城市基础设施向农村延伸，公共服务向农村覆盖，生产要素向农村辐射，促进城乡共同繁荣。
                           
                           　　进一步加快县域工业化进程。依托城市大工业，大力发展先进制造业及相关配套产业。突出发展食品及农副产品深加工业，全力开展农业产业化"双千提升行动"。大力推进县区合作共建，完善工业园区基础设施建设，着力培育县域工业规模企业群，做强园区经济、板块经济。
                           
                           　　继续加快农业战略性结构调整。着力建设一批"千棚连建"、"千亩连片"的产业基地；大力推进100个"一村一品"建设，打造一批产值超亿元的特色产业村；实施现代农业"百园示范工程"；积极推进畜牧升级计划和水产跨越工程，继续建设"千区万场"工程，努力形成特色成块、产业成带、集群发展的合肥现代农业产业新格局。
                           
                           　　深入推进基层农业社会化服务体系建设。实施"百社示范行动"，农民专业合作组织力争突破1000家，培育2-3家具有全省标杆和引领作用的农民专业合作社。强化农产品质量安全、农资、农民负担监管工作，不断提升服务农业的能力和水平。
                           
                           　　以土地综合整治为抓手，推动新一轮万亩土地复耕整理和整村推进新农村建设。完善推进农村土地承包经营权流转规范化管理和服务，流转农村土地100万亩，建设土地流转合作社100家。加强乡镇及村庄规划管理，完善小城镇基础设施建设。加快建设农村公路提级连网延伸工程，继续实施大中型病险水库除险加固、防洪保安和安全饮水工程。积极开展垃圾及污水处理、改厕等项目建设，大力推进秸秆综合开发利用，改善农村生产生活环境。
                           
                           　　（四）继续开展大建设，着力增强省会城市集聚辐射功能
                           
                           　　以实施合肥经济圈战略和"141"空间发展战略为引领，加快编制城市建设近期发展规划，提升区域整体实力和综合竞争力。
                           
                           　　继续强化综合交通枢纽建设。加快推进高铁南环线及合肥高铁站、宁西复线、合蚌、合福客运专线，争取商杭客运专线尽快开工，完成合肥火车站改建及北站货场建设。继续加快推进新桥国际机场建设。建成合肥港综合码头一期工程，加快推进巢湖复线船闸、裕溪复线船闸建设工程和合裕航线、派河航道整治工程，积极配合开展引江济巢工程前期工作。进一步完善城市出城口道路建设，开工建设机场高速、合店路（二期）、合白路，完成环巢湖道路路基工程，做好206国道、312国道改建前期工作。继续强化城市畅通能力建设，建成长江西路高架及轨道交通一号线试验段，基本完成裕溪路与南北一号线高架主体工程；完成二环改造、锦绣大道下穿桥建设，加快推进方兴大道与高速互通式出入口工程建设，进一步沟通"141"组团间交通联系；加强城市支线路网建设与改造，促进全市路网向环状加方格网转变。
                           
                           　　继续统筹推进四大组团建设，完善基础设施配套服务功能，提升建设品位和文化内涵。坚持"四个优先"，加大滨湖新区建设力度。加快总部经济、金融服务、旅游、会展、文化、商贸等基础设施建设；开展塘西河综合治理及巢湖岸线生态整治，打造滨水生态景观，加快中央公园、滨湖公园等建设。推进政务文化新区综合开发。充分发挥合肥大剧院、体育中心等平台作用，积极引进大型商业、服务业配套设施，建成青少年活动中心。
                           
                           　　坚持治理、建设并重，加快生态环境建设。完成二十埠河上游、板桥河上游及支流和十五里河河道的全面截污，推进小仓房、龚响塘二期等污水处理厂及配套管网建设，力争在全国率先实现污水全收集、全处理，基本实现"不让一滴生活污水流进巢湖"目标。推进生态补水工程建设，基本完成董铺水库溢洪道及南淝河生态补水工程。加快城市主干道绿化提质升级改造，初步建成一批园林景观大道。高标准建设城市生态廊道，巩固完善外环森林生态长廊。高品位实施公园景观建设，改造城区老公园，推进大蜀山森林公园建设。建设合淮阜、合六叶高速和合宁高铁两侧绿色长廊。出台扬尘污染防治办法和机动车尾气污染管理办法，实施机动车绿色环保标志管理。
                           
                           　　继续大力实施民生公用设施建设。加快推进拆迁安置复建点、廉租房等保障性住房建设，加大老城区、城中村、危旧房改造力度，提升市民居住水平。进一步加强水电气热等公用事业和市政基础设施建设，为市民提供高标准的公共服务。
                           
                           　　（五）加大体制机制创新力度，培育加快发展的新优势
                           
                           　　继续深化市级政府机构改革，切实解决部门及内部职能交叉问题。整合部门职能，稳步推进县区机构改革。推进城市管理体制改革，科学界定市、区两级权限，促进城市建设管理重心下移，强化城市管理。深化财政预算管理改革，加快建立"收支完整、相互衔接、有机统一、保障有力"的政府预算体系。积极稳妥推进工作用车使用管理改革。继续推进绩效预算评价制度，严格控制一般消费性支出，加大民生等重点支出保障力度。加强政府债务管理，推进政府融资平台与产业融资平台分离改革，进一步降低融资成本和风险，提高融资能力。
                           
                           　　全面加强国有资产监管，着力推动国有企业对外合作发展，拓宽国有资产管理领域，增强国有企业核心竞争力。推动国有企业深化内部改革，加快建立健全激励和奖惩机制，提升国有资本运营效率。继续推进事业单位绩效工资改革。继续深化节约集约用地试点市建设，坚持开源和节流并举，严格土地"一张图"管理、用地定额管理和"双向约束"制度，巩固土地清理成果，最大限度地发挥土地利用效益，形成顺畅有序的土地管理新机制。
                           
                           　　继续加大招商引资和对外开放力度。突出大产业、大项目，突出自主创新，突出提升城市服务功能，充分利用上海世博会、中国企业500强发布会等平台，强化重点区域、重点产业招商。落实出口退税、出口信用保险补贴、展位补贴等政策，支持企业开拓国际市场。加强区域合作，编制经济圈各项重点专项规划，有序推动合肥经济圈城市对接和互动发展，做好阜阳等皖北地区对口支持工作。继续开展国际友城交流与合作，做好外事、侨务、对台港澳工作。
                           
                           　　（六）加快发展现代服务业，提升区域产业层次和综合竞争力
                           
                           　　充分利用省会城市和区域中心城市的有利条件，以引进大项目和发展新型业态为重点，推进现代服务业提档升级。
                           
                           　　加快区域性金融中心建设。大力实施金融集聚工程，积极引进各类金融、保险机构总部、区域性总部、分支机构和后台服务中心；鼓励发展村镇银行、小额贷款公司等金融组织；加快发展金融租赁、典当、信托等非银行金融机构。整合招投标、文化、知识产权、金融期货、人力资源等要素市场，构建区域特色鲜明、带动辐射力强的要素大市场。完成科技农村商业银行增资扩股，做大做强兴泰控股等地方金融企业。创新银企对接方式，规范担保行业管理，发挥商会、行业协会等作用，推动以行业、产业链、供应链为基础，以核心企业为依托的融资对接活动。
                           
                           　　积极推动合肥经济圈文化旅游资源一体化开发，加快实现旅游一卡通和直通车；加快推进动漫主题公园、非物质文化遗产园、三国遗址公园、肥西圩堡群等项目建设，高标准推动安徽名人馆、渡江战役纪念馆建设，进一步打造三河、大圩、丰乐生态园等乡村文化旅游精品；加大培育扶持旅游航空市场力度，力争开通新的国际航线。制定会展业发展规划，推动滨湖国际会展中心建设，加大"国字号"展会争办力度。加快构筑合肥物流枢纽。以建设国家现代物流示范城市为契机，大力发展第三方物流，推动航空、新港、铁路货运枢纽物流园区及城际公路货运站场等重大项目建设，争取设立出口加工区和综合保税物流园区。积极推进国家服务外包示范城市建设，完善促进政策，培育领军企业，促进产业集聚。着力建设动漫产业基地，加快发展出版、文化娱乐、创意等产业。全面提升商贸服务业。继续实施"双进工程"、"万村千乡市场工程"和"新网工程"，加快周谷堆等农贸市场升级改造。大力建设和改造新老城区、中央商务区及一批特色商业街区，推广连锁经营、特许经营和新型业态。加强房地产市场运行分析，规范市场秩序，改善住房供应结构，提升物业管理水平，促进房地产市场平稳健康发展。
                           
                           　　（七）深入实施民生工程，促进社会事业全面发展
                           
                           　　继续围绕事关群众切身利益和社会和谐稳定的热点、难点问题，进一步加大民生投入，实施33项民生工程，促进经济社会协调发展。
                           
                           　　深入推进国家级创业型城市建设。放宽条件、扩大领域，坚决清理和禁止不合理收费，大力扶持初始创业，建设一批创业园、区、街和创业示范点；以"成长性小企业工程"为依托，大力支持中小企业、非公经济快速发展。加强高校毕业生就业见习基地建设；完善"零就业家庭"和困难群众再就业援助长效机制，全年新增城镇就业8万人。
                           
                           　　进一步推进社会保障体系建设。加快完善城镇职工、城市居民、新农合医疗保障制度，积极开展城乡居民医疗保障统筹试点；加快构建养老服务体系，规范散居"五保户"管理，积极开展新型农村养老和社区养老试点；切实做好困难群体、老年群体、优抚群体、残疾人群体等保障工作。进一步加快保障性住房建设，对符合申请廉租住房居民实行应保尽保。
                           
                           　　大力促进教育卫生事业健康发展。完善中小学布点规划，适时适度调整下划学校管理权限。大力实施中小学校舍安全工程和农村寄宿制学校建设工程，加大边远薄弱学校建设和改造力度。加快合肥职教城建设和市属职业学校整合力度，扶持发展优质民办教育，着力解决义务教育阶段择校收费、有偿家教及校园周边环境综合治理等问题。大力支持合肥学院建设省级示范应用型本科高校。加快医药卫生体制改革。实施农村乡镇卫生院、村卫生室标准化、一体化、信息化建设，加快社区卫生机构布局调整和"三主三辅"标准化建设；深化基层医药卫生体制改革，全面实施基本药物制度。
                           
                           　　继续推进文化体育事业繁荣发展。制定文化发展规划，建设市博物馆，精心打造一批文艺精品；继续实施"农家书屋"工程，建设一批标准化乡镇综合文化站。加大文化体制改革力度，推进国有文艺演出院团转企改制，完成市广播电视系统整合工作。
                           
                           　　积极承办第四届全国体育大会。围绕"赛、展、论、游"四大板块活动，突出"群众体育特点、合肥时代特色"，努力承办一次精彩圆满、令人难忘的体坛盛会。积极备战参赛省第十二届运动会，力争实现运动成绩和精神文明双丰收。
                           
                           　　统筹发展其他社会事业。切实做好人口和计划生育工作，稳定低生育水平。加快发展妇儿事业，确保实现"两纲"达标。抓好国防动员和后备力量建设，争创"双拥"七连冠。做好档案、地方志、未成年人保护、红十字会、气象和防震减灾等工作，重视并推动残疾人、慈善等事业发展。认真开展第六次全国人口普查。
                           
                           　　（八）继续开展城市文明创建，切实维护社会稳定
                           
                           　　深入推进精神文明创建活动。加强市民文明素养教育，弘扬文明新风，倡导文明行为。提升社区公共服务水平，推广社区网格化管理和标准示范社区建设，争创省、国家级和谐社区。扎实推进农村社区建设，力争全市10%以上的农村建制村转变为社区。继续推进"清洁家园、绿化乡村"活动。改进城市管理方式，提升管理水平，深入推进建筑垃圾运输密闭化管理改革，全面整治城市综合环境。
                           
                           　　切实加强社会治安综合治理。推进社会矛盾化解、社会管理创新、公正廉洁执法三项重点工作，依法打击各类违法犯罪活动，全力降低三类可防性案件，不断完善社会治安防控体系。继续推进法制城市创建，推行法律援助十项便民措施，争创全国"五五"普法先进城市，全面提升法治化管理水平。加大社会矛盾纠纷排查化解力度，提高突发性群体性事件处置能力。强化安全生产综合监管，严格落实安全生产责任，深入开展隐患排查治理。进一步落实信访工作领导责任制、责任追究制和包案责任制，继续深入开展领导干部定期接访和下行联合接访工作，下大力气解决一批疑难复杂信访问题。以保稳定、促发展、创和谐为主题，进一步加大民族宗教工作力度。提高民防、应急保障能力，扎实推进食品安全专项整治，严格药品安全监管，切实保障人民群众生命安全和身体健康。
 　　2010年是为"十二五"发展奠定基础至关重要的一年，在扎实抓好今年各项工作的同时，我们还要认真谋划，精心组织，加快编制完成"十二五"发展规划。
　　三、进一步加强政府自身建设
　　面对发展的新机遇、新要求，政府使命在肩、责任重大，必须进一步增强科学发展的紧迫感和责任感，切实加强自身建设，全面履行政府职能。
　　一是在进一步提高领导科学发展能力上下功夫。紧紧围绕加快发展这个主题，进一步提高公务员队伍整体素质，坚持做到抓好当前与谋划长远相结合、突出重点与统筹各方相结合、解决实际问题与建立长效机制相结合、关心弱势群体与维护大多数群众利益相结合，不断提高学习能力和科学认识市情能力，不断提高谋划科学发展能力，不断提高服务群众能力，不断提高解决问题能力，不断提高创造性工作能力。
　　二是在进一步建立长效机制上下功夫。继续推进体制机制创新，以制度巩固成果，用机制规范行为。高度重视人大代表议案、建议和政协委员提案办理工作，自觉接受人大及其常委会的监督，进一步发挥政协民主监督作用。进一步完善依法决策、科学决策、民主决策机制，建立健全重大决策调查研究、征求意见、专家论证、合法性审查、听证、公示等制度。加强和改进政府立法工作，扩大公众参与度，提高立法质量。创新问责机制，强化行政问责制度系统化建设。继续加快"数字合肥"建设，加大信息资源整合共享力度，推进政务信息公开，切实提高管理水平和公共服务能力。

　　三是在进一步转变职能改进政风上下功夫。深化行政审批制度改革，继续清理、取消和调整审批事项，强化后续和动态监管，建立行政效能电子监察系统。积极推进行政服务中心标准化建设，深化"两集中"和微观环节改革。深化行政执法体制改革，对部门内设及下属执法机构进行调整归并，规范现有行政执法类事业单位的职能，提高行政执法效能。坚持问政于民、问需于民，解决好人民群众最关心最直接最现实的利益问题。按照轻重缓急和难易程度，倒排解决问题时间表，做到既有近期计划，又有中长期安排。完善督办机制，按照责任分工，明确分管领导、分管部门，确保每一项措施有的放矢、对症下药，不落空、可操作。

　　四是在进一步加强反腐倡廉上下功夫。坚持标本兼治、综合治理、惩防并举、注重预防的方针，进一步加强执法监察、廉政监察、效能监察、审计监督和纠风治乱工作，加快推进惩治和预防腐败体系建设。加强对重点领域和关键环节的权力制约，特别要加强对领导干部、重点单位和关键岗位的监督和制约。开展商业贿赂专项治理，维护市场秩序和群众合法权益。坚持勤政廉政、自警自励，做遵纪守法的模范，做廉洁奉公的表率，以实际行动取信于民，营造风清气正的政治生态环境。
                           
                           　　各位代表！面对合肥加速崛起的新形势，我们唯有心无旁骛谋实干，殚精竭虑求发展，才能不辜负党和人民群众的重托。让我们更加紧密地团结在以胡锦涛同志为总书记的党中央周围，在省委、省政府和市委的坚强领导下，深入贯彻落实科学发展观，坚定信心，同心协力，攻坚克难，开拓创新，为继续保持合肥又好又快发展势头，加快现代化滨湖大城市建设而努力奋斗！
